# Soil Microbial Substrate Properties and Microbial Community Responses under Irrigated Organic and Reduced-Tillage Crop and Forage Production Systems

**DOI:** 10.1371/journal.pone.0103901

**Published:** 2014-08-04

**Authors:** Rajan Ghimire, Jay B. Norton, Peter D. Stahl, Urszula Norton

**Affiliations:** 1 Department of Ecosystem Science and Management, University of Wyoming, Laramie, Wyoming, United States of America; 2 Department of Plant Sciences, University of Wyoming, Laramie, Wyoming, United States of America; Catalan Institute for Water Research (ICRA), Spain

## Abstract

Changes in soil microbiotic properties such as microbial biomass and community structure in response to alternative management systems are driven by microbial substrate quality and substrate utilization. We evaluated irrigated crop and forage production in two separate four-year experiments for differences in microbial substrate quality, microbial biomass and community structure, and microbial substrate utilization under conventional, organic, and reduced-tillage management systems. The six different management systems were imposed on fields previously under long-term, intensively tilled maize production. Soils under crop and forage production responded to conversion from monocropping to crop rotation, as well as to the three different management systems, but in different ways. Under crop production, four years of organic management resulted in the highest soil organic C (SOC) and microbial biomass concentrations, while under forage production, reduced-tillage management most effectively increased SOC and microbial biomass. There were significant increases in relative abundance of bacteria, fungi, and protozoa, with two- to 36-fold increases in biomarker phospholipid fatty acids (PLFAs). Under crop production, dissolved organic C (DOC) content was higher under organic management than under reduced-tillage and conventional management. Perennial legume crops and organic soil amendments in the organic crop rotation system apparently favored greater soil microbial substrate availability, as well as more microbial biomass compared with other management systems that had fewer legume crops in rotation and synthetic fertilizer applications. Among the forage production management systems with equivalent crop rotations, reduced-tillage management had higher microbial substrate availability and greater microbial biomass than other management systems. Combined crop rotation, tillage management, soil amendments, and legume crops in rotations considerably influenced soil microbiotic properties. More research will expand our understanding of combined effects of these alternatives on feedbacks between soil microbiotic properties and SOC accrual.

## Introduction

Many changes in soil properties after conversion from one agricultural management system to another result from changes in soil microbiotic properties, defined here as the quality of microbial substrate and its effects on soil microbial communities [1], [2]. It is well known that management practices such as reduced-tillage, cover crops, and crop diversification increase soil microbial activity in general, and microbial biomass and diversity in particular [2]. Similarly, practices used in certified-organic food and feed production, including amendments and legume crops in rotations, support increased microbial biomass [1], [4], arbuscular mycorrhizal fungi (AMF) [5], and soil fauna [4]. It is not as clear how beneficial these management practices are under marginally productive conditions of cold, semiarid agroecosystems. In the study reported here, we evaluated whole-system effects on soil microbiotic properties after conversion from irrigated maize monoculture to conventional, reduced-tillage, and organic crop rotation systems in the central High Plains region of North America. Each management system combines a different suite of practices, including cultivation methods, crop rotations, and soil amendments.

Soil microbiotic properties are influenced by soil amendments, crop rotations, and tillage practices by different mechanisms. Organic amendments contribute diverse microbial substrates as heterogeneous organic materials in different states of decomposition [1], [4], while crop rotations diversify the supply of plant residues, including fine roots, root exudates, sloughed off tissues, and rhizodeposited materials, which drive diversification of soil microbial communities [2], [6], [7]. Intensive tillage drives pulses of microbial activity that mineralize labile soil organic matter (SOM) and shift microbiotic properties toward C-limited conditions that favor bacteria and reduce SOM concentrations, while reduced-tillage conserves labile substrates and creates a more consistent soil environment for microbial activity [2], [3], [8], [9]. In reduced-tillage systems, plant- and root-derived residues provide nucleation sites for fungal and bacterial growth, which further colonize soil particles to form aggregates and increase aggregate-protected, labile SOM and efficiency of substrate utilization (less C respired per unit of microbial biomass) [10], [11]. Perennial legume and non-legume forage crops in rotations further reduce soil disturbance compared with annual crops, and stimulate SOM accrual and microbial activity through increases in root biomass and residues [11]–[13]. Combinations of organic amendments and perennial legumes in rotations, which are common practices in certified organic crop and forage production, support more efficient soil N utilization than conventional, synthetic fertilizer-based management [4], [14] and can shift microbiotic properties toward N-limited conditions that favor fungi and accrue SOM.

Such management systems may be especially important in the central High Plains agroecosystem, where the semiarid environment, with inherently low SOM, cold winters, hot, dry summers, and irrigation-driven wetting-drying cycles, exacerbate mineralizing microbiotic conditions that drive losses of SOM [15]–[18]. Improved understanding of how reduced-tillage and organic crop and forage production systems affect soil microbial substrate quality, microbial biomass, community structure, substrate utilization, and soil organic C (SOC) sequestration in this cold and dry agroecosystem will help to design more sustainable systems during a time of uncertainty due to the changing climate, increasing operation costs, and changing markets [24]–[26].

The aim of this study was to evaluate SOC, DOC, C∶N ratios of microbial substrates, soil microbial biomass and community structure, and substrate utilization after transition from monocropped corn to crop rotations under conventional, organic, and reduced-tillage crop and forage production. The experiments were set up on inherently low fertility, irrigated soils in the dry and cold central High Plains agroecosystem. We hypothesized that crop rotations developed in the previously monocropped field would increase microbial biomass and microbial community diversity by increasing the quantity and changing the quality of microbial substrates. In addition, organic and reduced-tillage management systems would favor greater increases in soil microbial biomass and more diverse microbial communities with higher substrate utilization efficiency compared with conventional management.

## Materials and Methods

### Experimental Site

The four-year study was established in 2009 at the University of Wyoming Sustainable Agriculture Research and Extension Center (SAREC) near Lingle, Wyoming (42°7′15.03″N; 104°23′13.46″W). The study area has cool temperatures and a short growing season with an average frost-free period of about 125 days and 60-year average maximum and minimum temperature of 17.8°C and 0.06°C, respectively, and precipitation of 332 mm [19]. In addition, maximum and minimum air temperature and precipitation were monitored at the SAREC weather station within 1 km of the research plots during the study period. Monthly average maximum and minimum air temperature and monthly total precipitation throughout the study period are presented in Figure S1. Soil at the study site is mapped as Mitchell loam (loamy, mixed, active, mesic Ustic Torriorthent) with low SOM content (<1%), and slightly alkaline soil pH [20]. Soil texture of the study site was loamy with sand, silt and clay content of 41.0 (13.5)%, 41.4 (10.5)% and 17.6 (4.0)%, respectively (standard deviation in parentheses; n = 24).

### Experimental Design and Treatments

The study was designed as two independent randomized complete block experiments (row-crop production and forage production) laid out on a 15-ha half-circle under an irrigation pivot (305-m radius) that was divided into four wedge-shaped blocks (replications) (Figure S2). Each block was further separated into six plots consisting of three 0.405-ha crop production plots (outer three circles) and three 0.81-ha forage production plots (inner three circles). The three management-system treatments (conventional, certified organic, and reduced-tillage) were then randomly assigned to the crop and forage production plots. Before establishment of the experiment the entire area was under conventionally managed corn for at least six years.

All treatments were managed under four-year rotations starting in 2009. Table 1 shows the specific rotations, which were determined by a project advisory committee consisting of local producers and the SAREC management team. Under the conventional system inputs are applied as needed to maximize production, namely commercial synthetic fertilizer based on soil-test recommendations to supply nutrients, and chemical pesticides to control weeds, insects, and diseases. Specific management details are provided in Table S1. Conventional plots were moldboard ploughed, disked, and harrowed, which typically incorporates crop residues into soils leaving <15% of the soil surface covered by residues. The reduced-tillage system used conservation tillage that does not invert surface soil and leaves >15% residue cover on the soil surface. In the organic system, tillage was done as in conventional plots, and pest control and nutrient management were based on practices allowed by the USDA National Organic Program standards (http://www.ams.usda.gov/AMSv1.0/nop). Conventional and reduced-tillage systems had chemical weed and pest control.

**Table 1:** Crop rotations and management practices under different conventional (CV), organic (OR), and reduced-tillage (RT) management systems for crop and forage production (see Table S2 for detailed dates and management activities).

System		year	Crop in rotation	Management practices
Crop	CV	2009	Pinto bean	Tillage with moldboard plow and disk (5–7 passes each year), use of chemical fertilizers based on soil test recommendation for each crop, pesticides application as needed, and no livestock grazing.
		2010	Corn	
		2011	Sugar beet	
		2012	Corn	
	OR	2009	Alfalfa	Tillage with moldboard plow and disk (5–7 passes each year) and use of USDA-NOP certified practices for soil fertility (organic manure application) and pest management (e.g., cultivation), and no livestock grazing.
		2010	Alfalfa	
		2011	Corn	
		2012	Pinto bean	
	RT	2009	Pinto bean	Reduced-tillage (1–2 tillage passes each year that leave >15% crop residue on surface), use of chemical fertilizers based on soil test recommendation, pesticides application as needed, and no livestock grazing.
		2010	Corn	
		2011	Sugar beet	
		2012	Corn	
Forage	CV	2009	Alfalfa/grasses	Conventional tillage (5–7 passes in year 1 and 4), use of chemical fertilizers based on soil test recommendation, pesticides application as needed, and grazing with fall weaned calves during winter 2011/12.
		2010	Alfalfa/grasses	
		2011	Alfalfa/grasses	
		2012	Corn	
	OR	2009	Alfalfa/grasses	Conventional tillage and use of USDA certified practices for soil fertility (compost application) and pest management (no pesticides), and grazing with fall weaned calves during winter 2011/12.
		2010	Alfalfa/grasses	
		2011	Alfalfa/grasses	
		2012	Corn	
	RT	2009	Alfalfa/grasses	Reduced-tillage in the first year and no-tillage after, use of chemical fertilizers based on soil test recommendation, pesticides application as needed, and grazing with fall weaned calves during winter 2011/12.
		2010	Alfalfa/grasses	
		2011	Alfalfa/grasses	
		2012	Corn	

For soil fertility management, conventional and reduced-tillage plots received chemical fertilizer based on soil-test recommendations (Table S1). Organic management received composted cattle manure (dry matter 78% and C∶N∶P∶S = 24.6∶0.88∶0.22∶0.25%) in both crop and forage system in 2010. Because of the limited availability of composted cattle manure in 2011 and 2012, the organic crop system received raw manure (dry matter 29.2% and C∶N∶P∶S = 21.3∶1.42∶0.35∶0.40%) and the organic forage system received composted manure.

In the crop production experiment, management systems had different crops in rotation (Table 1). In forage system plots, a legume-grasses mixture was planted at 22 kg ha^−1^ in all plots in 2009, and included 50% alfalfa (*Medicago sativa* L.), 30% orchard grass (*Dactylis glomerata* L.), 10% meadow brome (*Bromus riparius* Rehmann), and 10% oat (*Avena sativa* L.) by weight. The forage production system plots were winter grazed for three months during 2011–2012 at stocking density of 1.6 fall-weaned calves ha^−1^.

### Soil Sampling

Soil samples were collected during spring, early summer, late summer, and fall seasons of the first (Year 1; 2009) and the fourth year (Year 4; 2012) from each of the 24 plots. During each sampling event, soil cores (3.2-cm diameter) were collected from 0–15 cm at 16 sampling points along a 50-m transect set in each plot, composited, thoroughly homogenized, subsampled (∼500 g), and placed on ice for transport to the laboratory. The 0–15 cm depth was considered to be sufficient because the focus was on near-surface microbial properties. Sampling transects were mapped using GPS (Trimble GeoXT, Sunnyvale, CA) to locate transects for subsequent sampling. In the laboratory, soil samples were stored at −20°C for PLFA analysis and at 4°C for DOC, TDN, and potential soil respiration. Phospholipid fatty acid contents in soil were analyzed within two weeks of soil sample collection. Soil bulk density was measured in a separate set of 2.1×15 cm cores collected from 8 sampling points along the 50-m transects. Soil samples from the first and the last sampling dates were analyzed for other soil properties described below.

### Laboratory Analysis

Total soil C and N were analyzed by dry combustion (EA1100 Soil C/N analyzer, Carlo Erba Instruments, Milan, Italy), inorganic C by modified pressure-calcimeter [21], and soil moisture by the gravimetric method [22]. Soil organic C was determined by subtracting inorganic C from total soil C. Soil pH was measured in a 1∶1 soil∶water mixture using an electrode [23]. Soil texture was determined by the hydrometer method [24]. Microbial substrate quality was determined as the ratio of DOC to total dissolved N (TDN) present in soils expressed as the C∶N ratio of microbial substrate. For this, 10 g of field-moist soil was extracted with 50 ml of 0.5 M K_2_SO_4_ and amounts of DOC and TDN were determined by 720°C combustion catalytic oxidation/chemiluminescence with a Schimadzu TOC Analyzer (TOC-VCPH with TNM-1, Schimadzu Scientific Instruments, Inc.) coupled with TOC-Control V Ver.2 analysis software. Dissolved inorganic C was removed by automatic acidification and sparging within the instrument. Potential soil respiration was determined as the amount of CO_2_-C mineralized during a two-week incubation period [25]. Soil bulk density was determined by the core method [26] and water filled soil pore space was calculated from bulk density and gravimetric moisture content [27].

Microbial biomass and community structure was analyzed by the Blight and Dyre [28] method of fatty acid methyl ester (FAME) analysis as modified by Frostegård et al. [29] and Buyer et al. [30]. Fatty acids were directly extracted from soil samples using a 1∶2∶0.8 chloroform∶methanol∶phosphate buffer mixture (0.15 *M*, pH 4.0), and PLFAs were separated from neutral and glycolipid fatty acids in a solid-phase extraction column (Agilent Technologies Inc.). The PLFAs were methylated using a mild methanoic KOH, and the FAMEs were analyzed using an Agilent 6890 gas chromatograph with autosampler, split-splitless injector (7683B series), and flame ionization detector (Agilent Technologies Inc.). The system was controlled with Agilent Chemstation and MIDI Sherlock software, and the fatty acid peaks were identified using the MIDI peak identification software (MIDI, Inc., Newark, DE, USA). All solvents and chemicals used were of analytical grade, and all glassware used was rinsed 10 times with deionized water, and sterilized overnight in 450°C in a Blue M lab heat box type muffle furnace (Blue M Electric, Richardson, TX). The PLFA signatures of 16 different fatty acids, which were quantified in almost all the field plots, were used to study soil microbial community structure and these fatty acids were grouped into gram positive, gram negative and other bacteria, AMF and other fungi, and protozoa (Table S3). In addition, the Shannon diversity index [31] was calculated as an index of soil microbial diversity as influenced by management systems in crop and forage production. The ratio of potential soil respiration to total PLFA microbial biomass was also calculated as an index of microbial substrate utilization.

### Statistical Analysis

Crop and forage production experiments were each analyzed as separate randomized complete block designs (RCBD) with three management-system treatments (conventional, organic, reduced-tillage) and four replicates. The analysis of soil properties that were measured at the beginning and end of the study, such as SOC, STN, pH and EC, were analyzed as split plot in time analysis of variance set in an RCBD for each system (p = 0.05). This analysis considered year as a repeated observation and replication as a random term in the model. Soil properties measured four times each year, such as soil microbial PLFA contents, DOC, C∶N ratio of microbial substrate, potential soil respiration, water filled pore space, and soil bulk density, were analyzed as a split plot in time analysis of variance that considered season and year as repeated observation terms in the model. Statistical computations for both designs were facilitated by the mixed model (Proc Mixed) procedure of the Statistical Analysis System (SAS, ver. 9.3, SAS Institute, Cary, NC). Means were separated using the PDIFF test in the LSMEANS procedure (p = 0.05) unless otherwise stated. There were no significant season×management system interactions for either system in the three way split plot in time analysis of variance, therefore, results are reported as average of all four seasons within a year. In addition, PLFA data for individual microbial groups were normalized to the total microbial PLFAs and the data (mole percent of total PLFAs) were reanalyzed through a multivariate method (principal component analysis) to compare shifts in microbial community structure. Relationships between soil microbial substrate properties, microbial biomass and community structure, and substrate utilization were analyzed using Pearson correlation. Principle component and Pearson correlation analyses were performed using a Minitab V.16.0 (Minitab Inc., State College, PA, USA) and the first two principal components are graphed to summarize the results.

## Results

Monthly average maximum and minimum temperatures during growing seasons (May to September) of 2009–2012 varied from year to year (Figure S1). The average minimum temperature was lowest in December 2009 and February 2011. Average precipitation was the lowest in 2012, followed by 2009 and 2010, compared to that in 2011. The amount of irrigation water depended on crop demand and the amount of precipitation received, and more water was applied to meet the crop water requirement in 2012 than in 2009–2011. All plots were irrigated to 60% of field capacity. Water filled porosity was consistent across management systems, seasons, and study years in both production systems (data not presented).

Soil pH was consistent across management systems and study years (range 7.3–7.8) under both crop and forage production, as was SOC concentration (Table 2). Soil organic C concentrations were, however, significantly influenced by a management system×year interaction. Soils under reduced-tillage (p = 0.034) and organic (p = 0.004) crop production had significantly more SOC than soils under conventional crop production. In addition, soils under organic crop production in the fourth year had significantly more SOC than in the first year (p = 0.02). Soils under reduced-tillage forage production had significantly more SOC than those under conventional forage production (p<0.01). Soil total N concentrations were not significantly influenced by management systems, years, or management system×year interactions under either crop or forage production. Soil bulk density was not significantly influenced by management system, season, or year, but was significantly influenced by a management system×year interaction under crop production. Specifically, soil bulk density was significantly higher under reduced-tillage than organic crop production in the fourth year (p = 0.007).

**Table 2:** Soil properties as influenced by conventional (CV), organic (OR), and reduced-tillage (RT) management systems for crop and forage production one and four years after transition from continuous conventional corn.

System		SOC† ‡			STN			DOC			Db		
		Y1	Y4	Δ%	Y1	Y4	Δ%	Y1	Y4	Δ%	Y1	Y4	Δ%
		g kg^−1^ soil			g kg^−1^ soil			mg kg^−1^ soil			g cm^−3^		
Crop	CV	6.70aA(1.31)	5.83bA(0.98)	−13.0	0.65(0.08)	0.73(0.12)	+12.3	19.5aB(2.08)	119cA(4.46)	+510	1.46aA(0.02)	1.43abA(0.02)	−2.05
	OR	6.52aB(0.16)	8.95aA(0.37)	+37.3	0.79(0.05)	0.90(0.08)	+13.3	36.4aB(5.45)	154aA(4.43)	+323	1.48aA(0.02)	1.40bA(0.02)	−5.41
	RT	7.31aA(0.86)	8.00aA(0.52)	+9.44	0.72(0.04)	0.80(0.05)	+11.8	20.6aB(3.78)	137bA(8.49)	+565	1.42aA(0.02)	1.48aA(0.03)	+4.23
Forage	CV	8.96aA(1.36)	7.23bA(0.46)	−19.3	0.77(0.09)	0.81(0.08)	+5.19	38.6aB(3.85)	139aA(13.3)	+360	1.47aA(0.03)	1.44aA(0.02)	−2.04
	OR	9.26aA(0.76)	8.50abA(0.35)	−8.31	0.77(0.02)	0.89(0.07)	+15.5	26.5aB(5.73)	147aA(7.36)	+455	1.47aA(0.02)	1.42aA(0.03)	−3.40
	RT	8.13aA(0.70)	10.2aA(1.04)	+25.5	0.86(0.14)	1.04(0.09)	+20.9	27.7aB(3.31)	152aA(2.67)	+449	1.39bA(0.02)	1.42aA(0.02)	+2.16

† Number in parenthesis indicates standard error.

‡ SOC = soil organic carbon, STN = soil total nitrogen, DOC = dissolved organic carbon (g kg^−1^ soil) and Db = soil bulk density (g cm^−3^). Different lowercase letters within a column indicate significant difference among management systems within a year and different uppercase letters indicate significant difference among years within a management system in crop as well as forage production (p = 0.05). No letter within a column or row indicates no significant management system×year difference.

**Figure 1:**
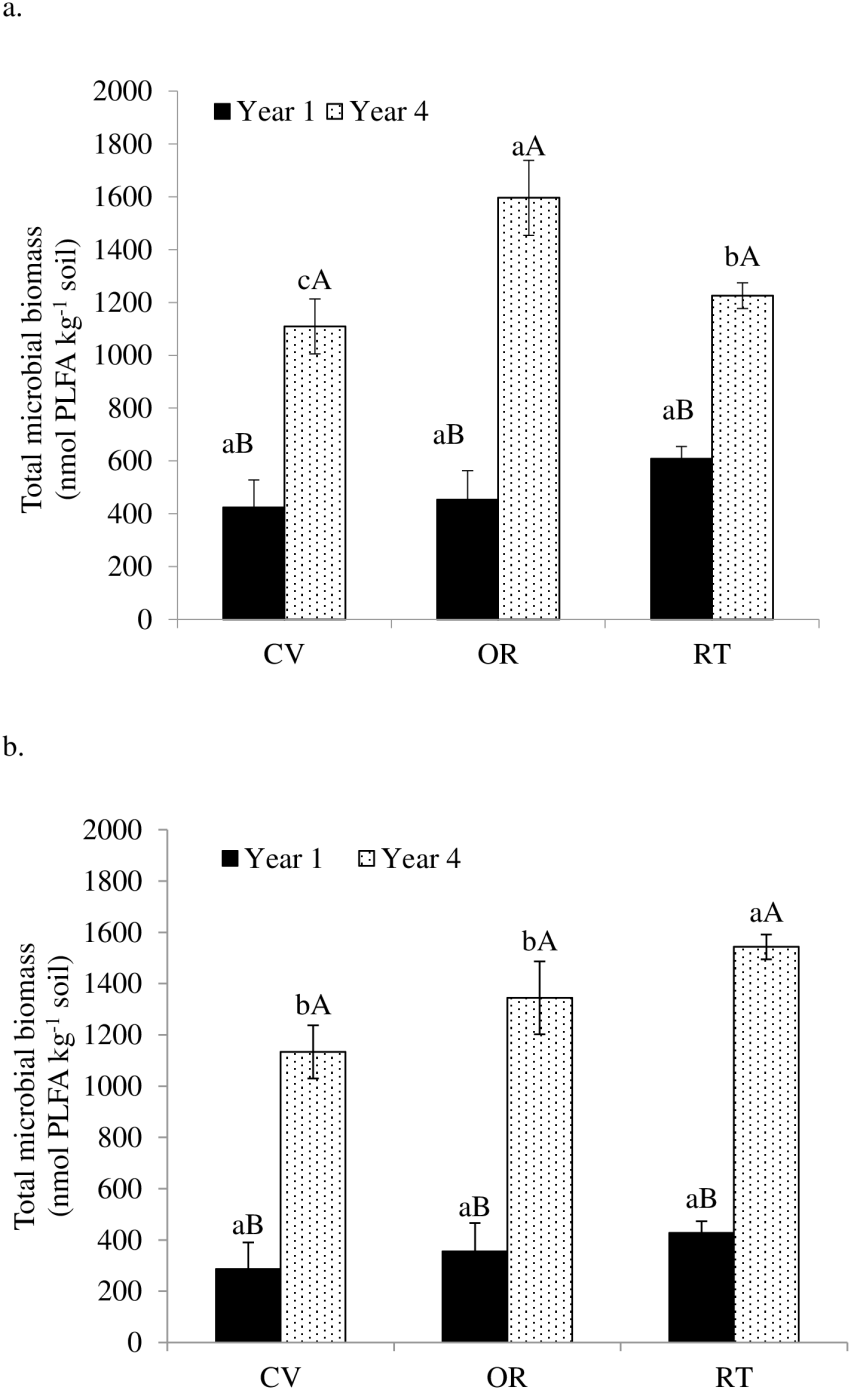
Total soil microbial biomass as influenced by conventional (CV), organic (OR), and reduced-tillage (RT) management systems for crop (a) and forage (b) production in the first and fourth years. Different lowercase letters indicate significant differences among management systems within a year and different uppercase letters indicate significant difference among years within a management system (p = 0.05).

Soil microbial biomass concentrations were significantly influenced by a management system×year interaction, but not by season. Specifically, in the fourth year under crop production there was significantly more soil microbial biomass under organic than conventional and (p<0.001) and reduced-tillage management (p = 0.01) (Fig. 1a). In the fourth year under forage production there was significantly more microbial biomass in soils under reduced-tillage than conventional (p = 0.002) and organic management (p = 0.047) (Fig. 1b). Under crop production, the greatest increase in total microbial biomass over the four-year period was observed in soils under organic management (353%) followed by conventional (262%) and reduced-tillage (202%) (based on year-one values). Under forage production, the increase in total microbial biomass concentrations were statistically similar at 396, 378 and 361% higher in the fourth year than in the first year in soils under conventional, organic, and reduced-tillage management systems, respectively.

**Figure 2:**
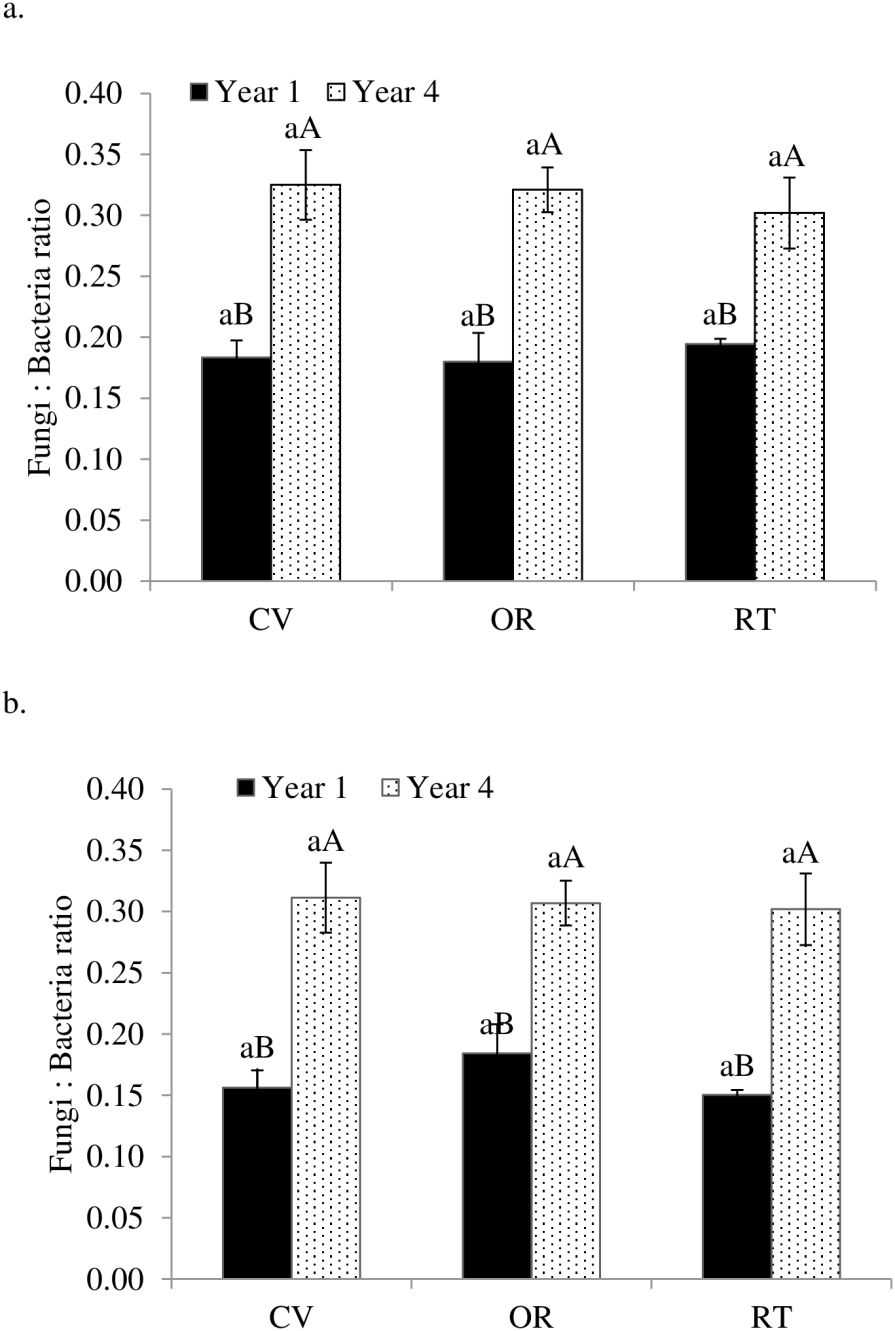
Fungal-to-Bacterial ratio as influenced by conventional (CV), organic (OR), and reduced-tillage (RT) management systems for crop (a) and forage (b) production in the first and fourth years. Different lowercase letters indicate significant differences among management systems within a year and different uppercase letters indicate significant difference among years within a management system (p = 0.05).

**Figure 3:**
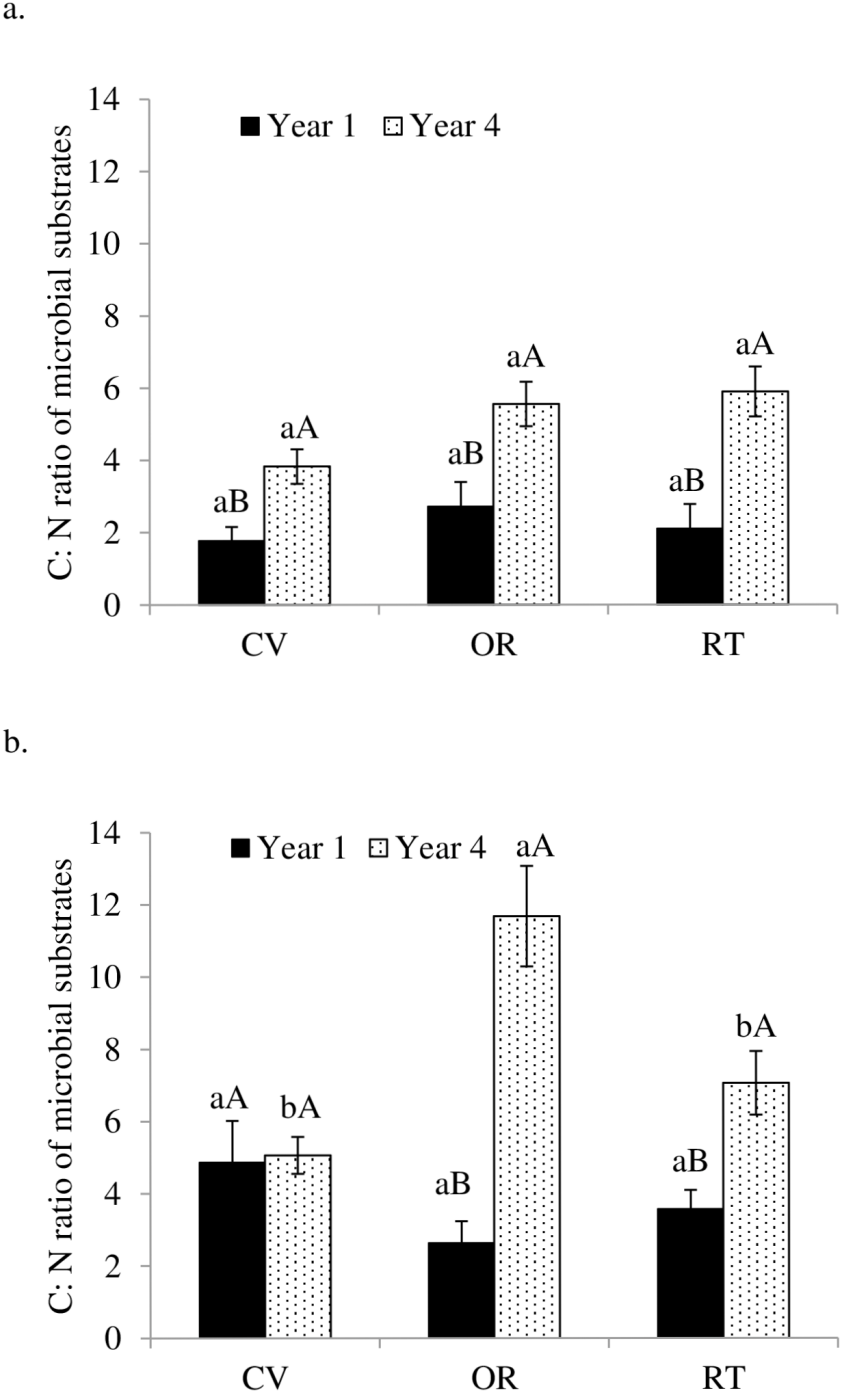
Carbon-to-nitrogen ratio of microbial substrate as influenced by conventional (CV), organic (OR), and reduced-tillage (RT) management systems for crop (a) and forage (b) production in the first and fourth years. Different lowercase letters indicate significant differences among management systems within a year and different uppercase letters indicate significant difference among years within a management system (p = 0.05).

**Figure 4:**
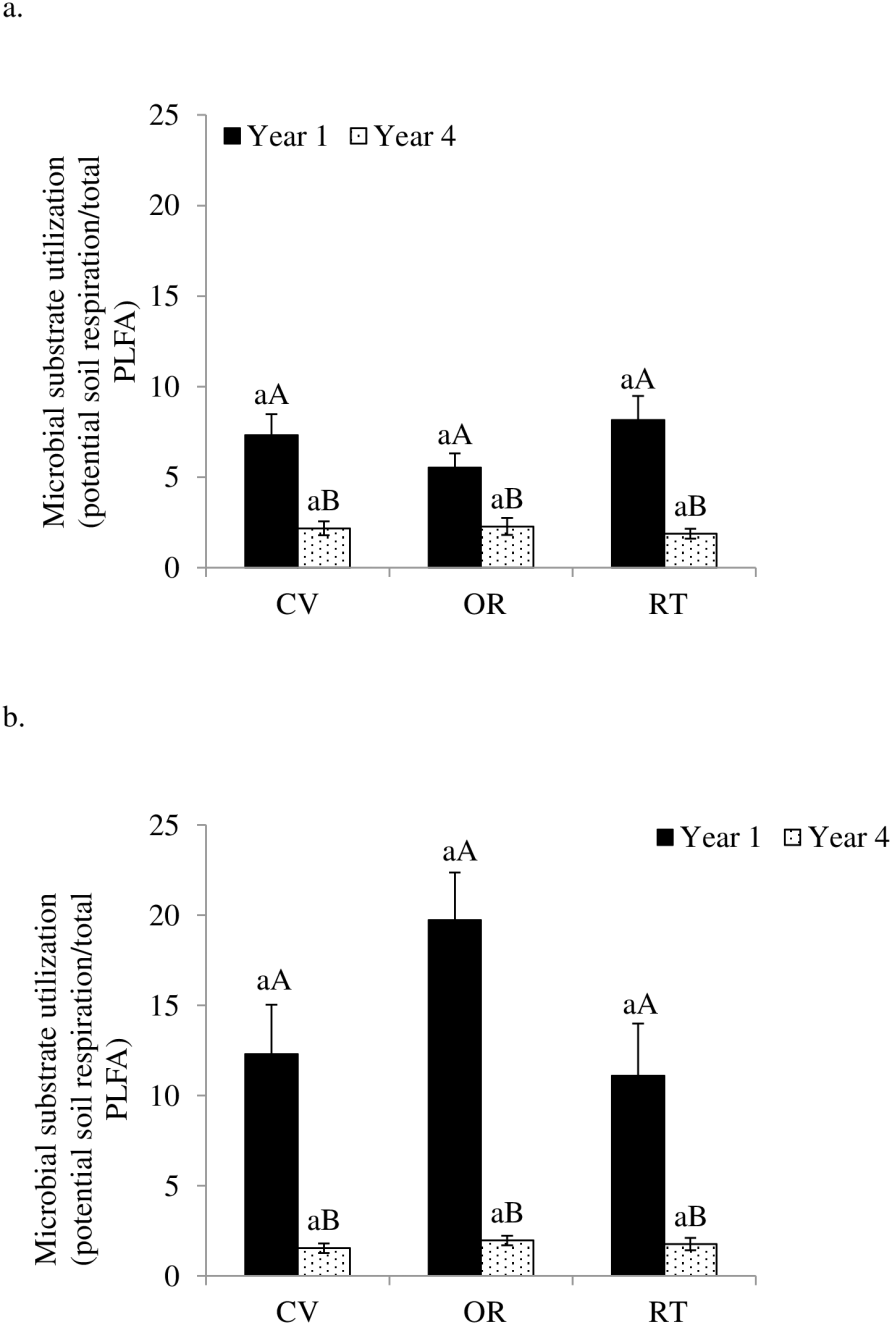
Microbial substrate utilization as influenced by conventional (CV), organic (OR), and reduced-tillage (RT) management systems for crop (a) and forage (b) production in the first and fourth years. Different lowercase letters indicate significant differences among management systems within a year and different uppercase letters indicate significant difference among years within a management system (p = 0.05).

We also observed increases in soil bacterial PLFAs, fungal biomarker PLFAs, DOC, and TDN across all treatments (only DOC data presented in Table 2), but the increases differed in magnitude. Dissolved organic C per unit SOC was 0.28–0.56% in the first year and 1.49–2.04% in the fourth year, with highest amount of DOC per unit SOC under conventional management. These changes corresponded with significantly higher fungal to bacterial ratios (F∶B ratios) (Figure 2) and C∶N ratios of microbial substrates (Figure 3) in the fourth year than in the first year. In addition, C∶N ratios of microbial substrates were greater in soils under organic forage production than under conventional and reduced-tillage forage production. Similarly, microbial substrate utilization (potential soil respiration per unit PLFA) was consistent across management systems (Figure 4), but was 85–90% lower under crop production and 61–77% lower under forage production in the fourth year than in the first year.

**Figure 5:**
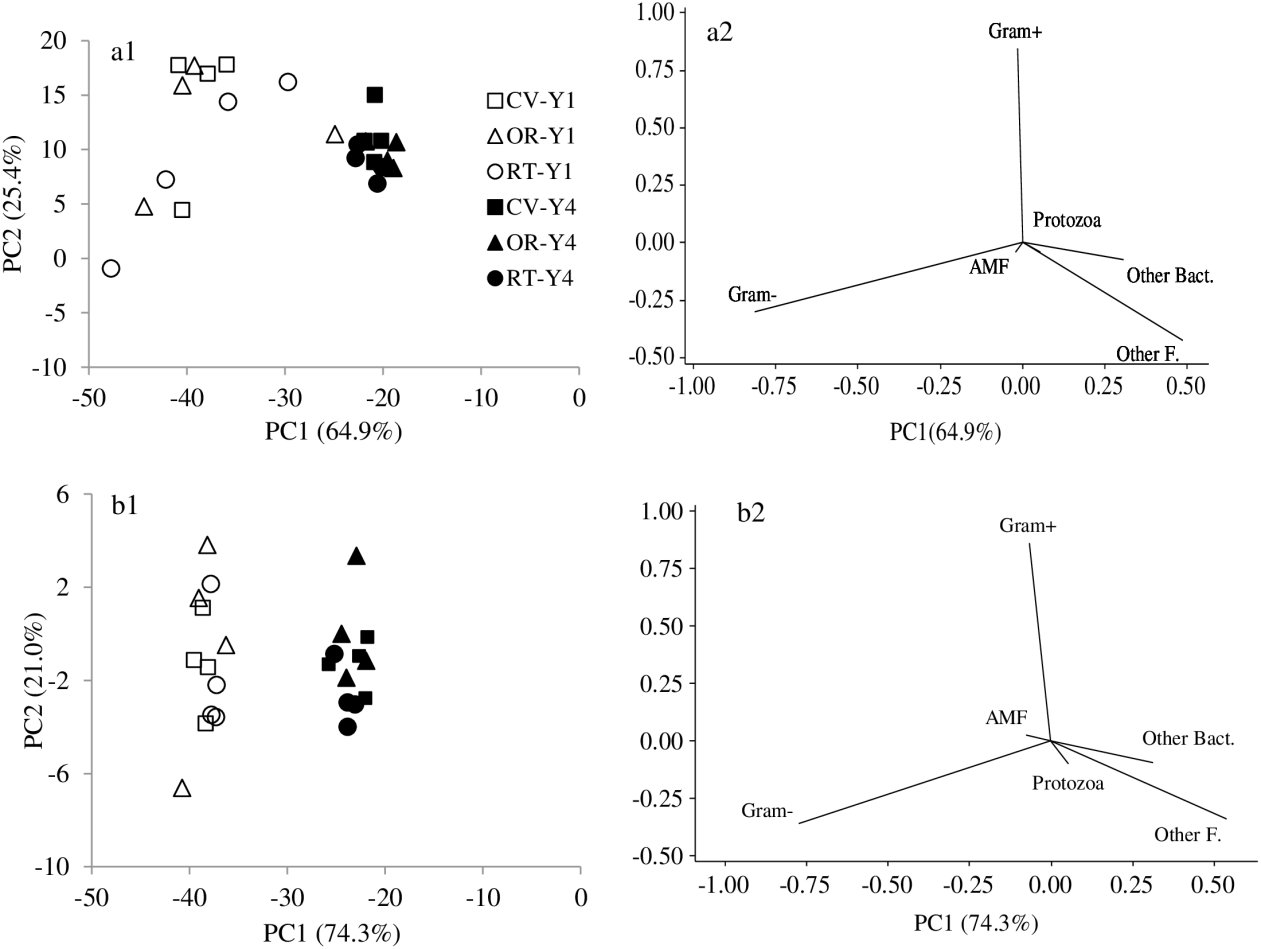
Score plots of the first two principle components (1) and loading of different microbial groups (2) as influenced by conventional (CV), organic (OR), and reduced-tillage (RT) management systems for crop (a) and forage (b) production. Gram+ = gram-positive bacteria, Gram− = gram-negative bacteria, AMF = arbuscular mycorrhizal Fungus, Other Bact. = other bacteria and Other F. = other fungus.

Principal component analysis of microbial community structure revealed that the first two principle components explained 64.9% and 25.4% of the total sample variance under the crop production, and 74.3% and 21.0% of total sample variance under the forage production (Figure 5). The soil samples collected in the first year clustered on the left side of the figures 5.a1 and b1, and those collected in the fourth year clustered on the right side, corresponding to the increase in microbial substrate quality and decrease in potential soil respiration. There was greater variance in microbial community data collected in the first year than in the fourth year under both crop and forage production. In addition, loading scores for management systems separated more clearly along PC2 in the fourth year than in the first year. Among microbial groups, protozoa, other bacteria, and other fungi had positive loadings, while AMF and gram-negative bacteria had negative loadings along the PC1 axis (Figure 5.a2 and b2). Along the PC2 axis, gram-positive bacteria under crop production, and both gram-positive bacteria and AMF under forage production, had positive loadings. Across management systems and crop and forage production, gram-positive bacteria, gram-negative bacteria, and AMF together constituted of 93% of total soil microbial biomass in the first year and 76–78% in the fourth year (Table 3). After four years under alternative management systems, biomarker PLFAs for these three microbial groups had increased 2–4 fold, while biomarker PLFAs for other bacteria, fungi, and protozoa had increased up to 26, 9, and 36 fold, respectively, corresponding with significant shifts in microbial community structure. Changes in microbial community structure over the four-year study period are also indicated by Shannon’s diversity index in Table 3, which was significantly higher in the fourth year than in the first year across both crop and forage production and all three management systems.

**Table 3:** Soil microbial communities as influenced by conventional (CV), organic (OR), and reduced-tillage (RT) management systems for crop and forage production one and four years after transition from continuous conventional corn.

System		Gram positivebacteria†			Gram negativebacteria			Other Bacteria			AMF				Other Fungi			Protozoa			Shannon’s diversity index		
		Y1	Y4	Δ%	Y1	Y4	Δ%	Y1	Y4	Δ%	Y1	Y4		Δ%	Y1	Y4	Δ%	Y1	Y4	Δ%	Y1	Y4	Δ%
		nmol kg^−1^ soil			nmol kg^−1^ soil			nmol kg^−1^ soil			nmol kg^−1^ soil				nmol kg^−1^ soil			nmol kg^−1^ soil					
Crop	CV‡	200aB(53.3)	414bA(43.7)	+107	160bB(33.8)	391cA(60.4)	+144	12.2aB(5.64)	60.3bA(11.7)	+394	28.7aB(6.21)		76.5bA(7.24)	+167	20.0bB(5.01)	152bA(15.8)	+665	3.30(1.81)	14.4bA(2.70)	+336	3.50aB(0.25)	5.53aA(0.23)	+58.2
	OR	213aB(51.6)	555aA(48.8)	+161	166bB(34.4)	559aA(84.4)	+237	12.0aB(4.26)	105aA(18.9)	+775	28.8aB(7.00)		118aA(13.9)	+310	29.1aB(6.70)	230aA(16.1)	+201	3.02(1.63)	28.7aA(2.17)	+850	3.55aB(0.29)	5.89aA(0.20)	+66.0
	RT	270aB(60.7)	467bA(54.1)	+72.9	236aB(45.9)	479bA(78.2)	+106	22.4aB(6.97)	70.9bA(13.5)	+217	44.3aB(8.88)		89.6bA(10.5)	+116	31.7aB(7.17)	173bA(23.0)	+147	4.38(2.36)	21.4abA(3.28)	+754	3.80aB(0.27)	5.71aA(0.27)	+50.2
Forage	CV	124aB(21.5)	391bA(23.7)	+215	117aB(17.5)	408bA(55.1)	+249	2.30aB(1.61)	51.5aA(10.6)	+2139	24.9aB(3.50)		86.7bA(7.99)	+248	18.2aB(3.59)	176aB(15.8)	+867	0.00	21.1(2.31)	-	3.85aB(0.22)	5.69aA(0.20)	+47.9
	OR	157aB(22.3)	474abA(55.3)	+197	142aB(18.8)	490bA(93.0)	+245	3.69aB(2.26)	70.7aA(11.5)	+1575	29.5aB(3.64)		111aA(22.7)	+276	23.0aB(3.77)	177aB(16.1)	+670	0.00	22.8(3.87)		4.00aB(0.16)	5.64aA(0.22)	+41.0
	RT	180aB(24.2)	526aA(41.6)	+192	177aB(19.5)	566aA(72.1)	+219	3.11aB(2.19)	80.4aA(19.4)	+2485	36.9aB(4.22)		116aA(12.3)	+214	29.1aB(3.30)	218aB(23.0)	+649	1.13(1.13)	37.1(2.94)	+3597	4.19aB(0.04)	5.71aA(0.22)	+36.1

† Number in parenthesis indicates standard error.

‡ AMF = Arbuscular Mycorrhizal Fungi. Different lowercase letters within a column indicate significant difference among management systems within a year and different uppercase letters indicate significant difference among years within a management system in crop as well as forage production (p = 0.05). No letter within a column or row indicates no significant management system×year difference.

Increases in microbial biomass and F∶B ratios, along with other changes in microbial community structure, along PC1 were strongly positively correlated with substrate availability (DOC concentrations) and quality (C∶N ratio of microbial substrate) (Table 4) under both crop and forage production. Microbial substrate utilization decreased significantly with increasing substrate availability, increasing C∶N ratios of microbial substrates, and increasing microbial biomass. Similarly, microbial community changes along PC2 were not related with substrate properties and substrate utilization.

**Table 4:** Significant correlation coefficients (r) between soil microbial substrate properties, microbial community and substrate utilization in crop and forage production systems.

	DOC† ‡	C∶N MAS	Microbial biomass	F∶B ratio	PC1§
**Crop system**					
Substrate C∶N	0.69(<0.001)	-			
Microbial biomass	0.83(<0.001)	0.86(<0.001)	-		
F∶B ratio	0.83(<0.001)	0.81(<0.001)	0.91(<0.001)	-	
PC1	0.78(<0.001)	0.63(0.001)	0.83(<0.001)	0.91(<0.001)	-
Soil Resp.	−0.64(0.001)	−0.47(0.02)	−0.70(<0.001)	−0.74(<0.001)	−0.84(<0.001)
**Forage system**					
Substrate C∶N	0.65(0.001)	-			
Microbial biomass	0.85(<0.001)	0.56(0.006)	-		
F∶B ratio	0.91(<0.001)	0.64(0.001)	0.83(<0.001)	-	
PC1	0.93(<0.001)	0.67(0.001)	0.91(<0.001)	0.97(<0.001)	-
Soil Resp.	−0.79(<0.001)	−0.40(<0.001)	−0.87(<0.001)	−0.73(<0.001)	−0.84(<0.001)

† Number in parenthesis indicates Pearson correlation p values.

‡ DOC = dissolved organic carbon, F∶B ratio = fungi to bacteria ratio, PC1 = First principal component and Soil Resp. = potential soil respiration.

§ PC1 explains shift in soil microbial community structure from the first to the fourth year.

## Discussion

Our results support our hypotheses and indicate that conversion from continuous corn to crop rotations positively impacted soil microbiotic properties across all three management systems, with higher substrate availability, substrate C∶N ratios, and soil microbial biomass contents, but lower substrate utilization in the fourth year than in the first year following transition (Tables 2 and 4; Figures 1a, 3a, and 4a). Both reduced-tillage and organic management systems added to these effects.

Under organic crop production, combined effects of perennial legumes in the rotations, which eliminated tillage for two of the four years, with additions of manure and compost, apparently offset losses of microbial substrates due to heavy tillage during the annual crop phases and supported the highest year-four microbial biomass concentrations (Table 2). Inclusion of legumes in rotations and organic amendments typically favor microbial growth, SOC and N accumulation, and diversification of microbial substrates [6], [7], [33]. Under reduced-tillage crop production, a more consistent soil environment apparently facilitated higher soil microbial biomass concentrations and diversity, as well as higher fungal productivity than under conventional management (Table 3; Figure 1a). Similar effects of reduced disturbance have been noted [2], [9], [34] in which fungal hyphae improve soil aggregation, which protects labile SOM components and regulates microbial substrate utilization [3], [12], [13], [33].

Under forage production, reduced-tillage management had the highest year-four microbial biomass concentrations of the three systems, probably due to lack of plowing with conversion from perennial forage to corn. Organic management, with applications of composted manure, created significantly higher C∶N ratios of microbial substrates in the fourth year than under conventional and reduced-tillage with chemical fertilizer application (Figure 3b). The fact that this difference in substrate quality did not occur under the crop production experiment (Figure 3a) suggests that it resulted from a combined effect of the alfalfa-grasses mixture and compost applications. In the conventional and reduced-input forage systems, N from chemical fertilizer and alfalfa may have contributed to lower substrate C∶N ratios than under the organic forage system.

Changes in soil microbiotic properties we observed in response to alternative management systems are consistent with results of previous studies, but greater in magnitude. Previous studies have reported two- to three-fold increases in microbial biomass with diversified rotations or reduced disturbance in place for several years [2], [8], [32], [35]. The greater magnitudes we observed probably resulted from combined effects of transition from continuous corn to rotations with legumes, manure applications, and reduced-tillage on depleted, inherently-low-fertility soils. While variable precipitation during the seasons of the study may have influenced comparisons between years 1 and 4, air temperatures (Figure S1) and soil water filled pore space were consistent among management systems across years under both crop and forage production. Therefore, we believe that the changes in management, rather than annual climatic variability, drove the observed changes in soil microbiotic properties.

The changes in quantity and quality of microbial substrates during the study period drove notable shifts in microbial community structure, including greater increases in fungal relative to bacterial PLFAs (Table 3; Figure 2), in gram-positive relative to gram-negative bacterial PLFAs, and in saprophytic fungi and protozoa relative to other groups. Higher DOC and C∶N ratios of microbial substrates at the end of the study drove greater increases in saprophytic fungi, which rely on carbonaceous substrates, than AMF, which are often associated with more mineral-rich, low C∶N substrates [36]–[38]. The observed increases in the non-mycorrhizal fungi strongly correlated with increased substrate availability, indicating changes in C∶N ratios favored more fungal growth (saprotrophic fungi) than bacterial growth.

Increases in gram-positive relative to gram-negative bacteria are also often associated with increases in diversity of C sources in soils and decreases in mechanical soil disturbance, [36]–[39]. Gram-positive bacteria are associated with low substrate availability (high C∶N) environments, while gram-negative bacteria dominate soils with more easily decomposable substrates [38]. Decreases in relative abundance of gram-negative bacteria may be beneficial because many plant pathogenic microorganisms such as *Pseudomonas* and *Xanthomonas* species are gram negative [40], [41]. Large increases in protozoa parallel the increases soil bacteria, which are their food source [42].

**Figure 6:**
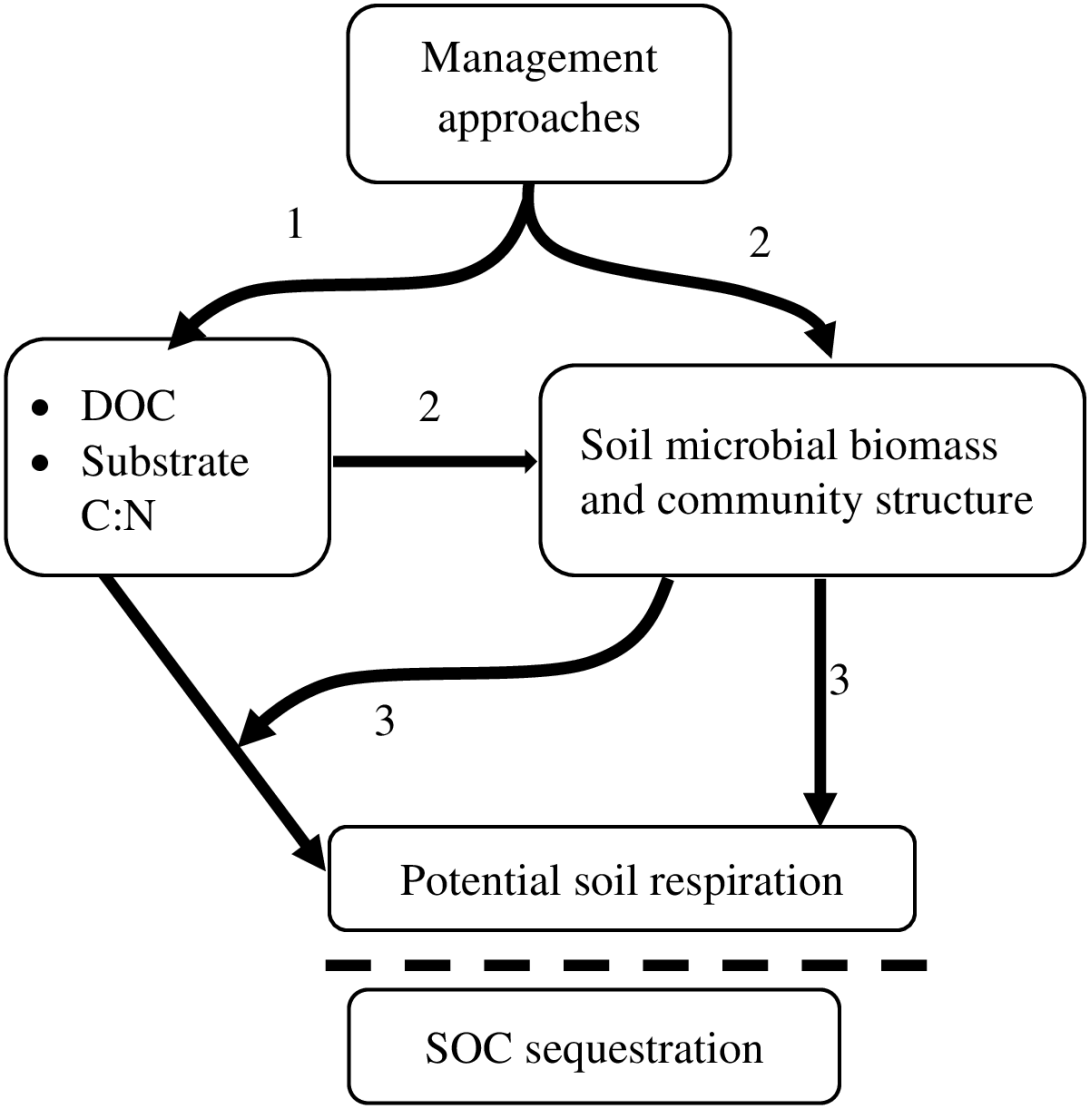
Conceptual framework illustrating influence of management system on microbial substrate properties, microbial communities, and SOC sequestration. Alternative management systems influence (1) microbial substrate availability and quality, (2) soil microbial biomass and community structure, and (3) soil respiration and SOC sequestration. DOC = dissolved organic carbon (microbial substrate).

Greater amounts of higher C∶N-ratio microbial substrates, more diverse communities of microorganisms with higher F∶B ratios, and reduced potential soil respiration in the fourth year across all three management systems in general, and under reduced-tillage forage and organic crop systems in particular, suggests that minimum soil disturbance, application of organic amendments, and more legume crops in rotation increase soil microbial biomass, alter community structure, and thereby influence SOC accrual (Figure 6). Although mechanisms of SOC regulation by specific groups of microorganisms are not well defined, given similar site characteristics, higher SOC sequestration potential is typically observed in soils with higher F∶B ratios [43], [44]. Higher SOC in fungal-dominated systems is mainly attributed to higher biomass C production per unit of C metabolized by fungus than by soil bacteria [44]. The changes in microbiotic properties we observed indicate that substrate C was mainly transformed into microbial biomass or less labile SOM components, which may be reflected in year-four SOC contents.

Under crop production, both our organic system, with two years of alfalfa, and reduced-tillage system involved considerably less soil disturbance than our conventional system, and both had more year-4 SOC than the conventional system (Table 1, S1). Under forage production, with the same rotation across the three management systems, reduced tillage had significantly more year-4 SOC than the other two system, indicating that organic amendments combined with intensive tillage did not increase SOC. Taken together, these results suggest that reduced-tillage combined with legumes in rotation had the largest impacts on SOC accrual. Repeated tillage to plow down the grasses and alfalfa and establish corn in year 4 might have caused significant loss of SOC accrued during three years under forages in both organic and conventional systems.

Overall, increases in microbial substrate availability and microbial biomass over the 4-year study represent a small fraction of SOC reservoirs, even in this low-SOM environment. Therefore, longer-term evaluation of the effects of tillage, crop rotations, soil amendments, and legume integration in crop and forage production may further our understanding of the influence of microbiotic properties on SOC sequestration. Results of this cropping systems study bundle effects of reduced tillage, crop rotation, legumes in rotation, and soil fertility options into three management systems for crop and forage production. While overall effects are crucial to understanding how management alternatives affect system sustainability, evaluating individual components will complement these results and contribute to design of best management practices for irrigated agriculture in cold, semiarid agroecosystems like the central High Plains.

## Conclusions

In this study, management systems that included reduced tillage, perennial legumes, and organic amendments improved soil microbiotic properties that support SOC accrual. The greatest changes occurred with transition from continuous corn to crop rotation. The changes were enhanced under organic management in cash-crop production and reduced-tillage management under forage production. Under the different rotations of our crop production systems, more legume crops in rotation had greater influence on soil microbiotic properties than fewer legume crops. Under the same rotations of our forage production systems, reduced-tillage management had the greatest influences on soil microbiotic properties. These effects were driven by interactions between soil microbial community structure and microbial substrate quantity and quality that resulted in increases in fungal biomass that support SOC accrual. The results indicate that reducing disturbance, including legumes, and applying organic amendments positively impact soil processes in ways that enhance sustainable productivity of inherently low-fertility soils in a cold, semiarid environment, even over a relatively short time period. Further research may confirm the combined effects of crop rotations and alternative management systems on SOC accrual and ecosystem services as influenced by soil microbial biomass and fungal productivity.

## Supporting Information

Figure S1 Monthly average maximum and minimum temperature, and total precipitation at the SAREC weather station, Lingle, Wyoming (Apr. 2009–Sept. 2012).
(TIFF)

Figure S2 Experimental design of crop and forage production plots under alternative management systems (C, conventional; O, organic; R, reduced tillage). Tiers A, B, and C are 1-acre cash-crop plots; tiers D, E, and F are 2-acre forage plots.
(TIFF)

Table S1 Crop rotations under conventional (CV), organic (OR) and reduced-tillage (RT) management systems in crop and forage production.
(DOCX)

Table S2 Timing of management practices by under conventional (CV), organic (OR), and reduce-tillage (RT) management systems on crop and forage production (2009–2012).
(DOCX)

Table S3 Biomarker phospholipid fatty acids used for identifying taxonomic microbial groups.
(DOCX)

## References

[1] Berthrong ST, Buckley DH, Drinkwater LE (2013) Agricultural management and labile carbon additions affect soil microbial community structure and interact with carbon and nitrogen cycling. Microbial Ecol 66: 158–170. 10.1007/s00248-013-0225-0 23588849

[2] Acosta-Martinez V, Mikha MM, Vigil MF (2007) Microbial communities and enzyme activities in soils under alternative crop rotations compared to wheat-fallow for the Central Great Plains. Appl Soil Ecol 37: 41–52.

[3] Stahl PD, Parkin TB, Christensen M (1999) Fungal presence in paired cultivated and undisturbed soils in central Iowa. Biol Fertil Soils 29: 92–97.

[4] Drinkwater LE, Letourneau DK, Workneh F, Vanbruggen AHC, Shennan C (1995) Fundamental differences between conventional and organic tomato agroecosystems in California. Ecol Appl 5: 1098–1112.

[5] Oehl F, Sieverding E, Ineichen K, Mader P, Boller T, et al (2003) Impact of land use intensity on the species diversity of arbuscular mycorrhizal fungi in agroecosystems of Central Europe. Appl Environ Microbiol 69: 2816–2824. 12732553 10.1128/AEM.69.5.2816-2824.2003 PMC154529

[6] Kandeler E, Tscherko D, Spiegel H (1999) Long-term monitoring of microbial biomass, N mineralisation and enzyme activities of a Chernozem under different tillage management. Biol Fertil Soils 28: 343–351.

[7] Shi Y, Lalande R, Ziadi N, Sheng M, Hu Z (2012) An assessment of the soil microbial status after 17 years of tillage and mineral P fertilization management. Appl Soil Ecol 62: 14–23.

[8] Acosta-Martínez V, Dowd SE, Bell CW, Lascano R, Booker JD, et al (2010) Microbial community structure as affected by dryland cropping systems and tillage in a semiarid sandy soil. Diversity 2: 910–931.

[9] Halvorson AD, Wienhold BJ, Black AL (2002) Tillage, nitrogen, and cropping system effects on soil carbon sequestration. Soil Sci Soc Am J 66: 906–912.

[10] Blanco-Canqui H, Lal R (2007) Regional assessment of soil compaction and structural properties under no-tillage farming. Soil Sci Soc Am J 71: 1770–1778.

[11] Ghimire R, Norton J, Pendall E (2014) Alfalfa-grass biomass, soil organic carbon, and total nitrogen under different management systems in an irrigated agroecosystem. Plant Soil 374: 173–184.

[12] Six J, Feller C, Denef K, Ogle SM, Sa MJC, et al (2002) Soil organic matter, biota and aggregation in temperate and tropical soils: Effects of no-tillage. Agronomie 22: 755–775.

[13] Liebig M, Carpenter-Boggs L, Johnson JMF, Wright S, Barbour N (2006) Cropping system effects on soil biological characteristics in the Great Plains. Renew Agric Food Syst 21: 36–48.

[14] Jenkinson DS, Parry LC (1989) The nitrogen-cycle in the broadbalk wheat experiment - a model for the turnover of nitrogen through the soil microbial biomass. Soil Biol Biochem 21: 535–541.

[15] Ghimire R, Norton JB, Norton U, Ritten JP, Stahl PD, et al (2013) Long-term farming systems research in the central High Plains. Renew Agric Food Syst 28: 183–193.

[16] Krall JM, Delaney RH, Taylor DT (1991) Survey of nonirrigated crop production practices and attitudes of Wyoming producers. J Agron Educ 20: 120–122.

[17] Norton JB, Mukhwana EJ, Norton U (2012) Loss and recovery of soil organic carbon and nitrogen in a semiarid agroecosystem. Soil Sci Soc Am J 76: 505–514.

[18] Frey SD, Elliott ET, Paustian K (1999) Bacterial and fungal abundance and biomass in conventional and no-tillage agroecosystems along two climatic gradients. Soil Biol Biochem 31: 573–585.

[19] Western Regional Climate Center (2013) Historical climate information. Reno, NV: Desert Research Institute. http://www.wrcc.dri.edu/. Accessed: 06-15, 2013.

[20] Soil Survey Staff (2013) Web Soil Survey. Natural Resources Conservation Service, United States Department of Agriculture. http://websoilsurvey.sc.egov.usda.gov/ Accessed: 06-15, 2013.

[21] Sherrod LA, Dunn G, Peterson GA, Kolberg RL (2002) Inorganic carbon analysis by modified pressure-calcimeter method. Soil Sci Soc Am J 66: 299–305.

[22] Gardner WH (1986) Water content. In: Klute A, editor. Methods of Soil Analysis Part 1: Physical and Mineralogical Methods. 2nd ed. Madison, WI: Agronomy Monograph 9. ASA, SSSA pp. 493–541.

[23] Thomas GW (1996) Soil pH and soil acidity. In: Sparks DL, editor. Methods of Soil Analysis, part 3: Chemical Methods. Madison, WI: Agronomy Monograph 9. ASA, SSSA pp. 475–490.

[24] Gee GW, Bauder JW (1986) Particle-size analysis. In: Klute A, editor. Methods of Soil Analysis Part 1: Physical and Mineralogical Methods. 2nd ed. Madison, WI: Agronomy Monograph 9. ASA, SSSA pp. 383–411.

[25] Nie M, Pendall E, Bell C, Gasch CK, Raut S, et al (2012) Positive climate feedbacks of soil microbial communities in a semi-arid grassland. Ecol Letters 16: 234–241. 10.1111/ele.12034 23157642

[26] Blake GR, Hartge KH (1986) Bulk density. In: Klute A, editor. Methods of Soil Analysis, part 1: Physical and Mineralogical Methods. 2nd ed Madison, WI. ASA, SSSA 363–375.

[27] Linn DM, Doran JW (1984) Effect of water-filled pore-space on carbon-dioxide and nitrous-oxide production in tilled and nontilled soils. Soil Sci Soc Am J 48: 1267–1272.

[28] Blight EG, Dyre WJ (1959) A rapid method of total lipid extraction and purification. Can J Biochem Physiol 37: 911–917. 13671378 10.1139/o59-099

[29] Frostergard A, Tunlid A, Baath E (1991) Microbial biomass measured as total lipid phosphate in soils of different organic content. J Microbiol Methods 14: 151–163.

[30] Buyer JS, Roberts DP, Russek-Cohen E (2002) Soil and plant effects on microbial community structure. Can J Microbiol 48: 955–964. 12556123 10.1139/w02-095

[31] Shannon CE, Weaver W (1949) The Mathematical Theory of Communication. Urbana, IL: Univ. of Illinois Press. 132 p.

[32] Minoshima H, Jackson LE, Cavagnaro TR, Sanchez Moreno S, Ferris H, et al (2007) Soil food webs and carbon dynamics in response to conservation tillage in California. Soil Sci Soc Am J 71: 952–963.

[33] Delate K, Cambardella CA (2004) Agroecosystem performance during transition to certified organic grain production. Agron J 96: 1288–1298.

[34] Ngosong C, Jarosch M, Raupp J, Neumann E, Ruess L (2010) The impact of farming practice on soil microorganisms and arbuscular mycorrhizal fungi: Crop type versus long-term mineral and organic fertilization. Appl Soil Ecol 46: 134–142.

[35] Reganold JP, Andrews PK, Reeve JR, Carpenter-Boggs L, Schadt CW, et al (2010) Fruit and soil quality of organic and conventional strawberry agroecosystems. Plos One 5: e12346. 20824185 10.1371/journal.pone.0012346 PMC2931688

[36] Fliessbach A, Oberholzer HR, Gunst L, Maeder P (2007) Soil organic matter and biological soil quality indicators after 21 years of organic and conventional farming. Agric Ecosyst Environ 118: 273–284.

[37] Carpenter-Boggs L, Stahl PD, Lindstrom MJ, Schumacher TE, Barbour NW (2003) Soil microbial properties under permanent grass, conventional tillage, and no-till management in South Dakota. Soil Till Res 71: 15–23.

[38] Fierer N, Schimel JP, Holden PA (2003) Variations in microbial community composition through two soil depth profiles. Soil Biol Biochem 167–176.

[39] Schaad NW, Jones JB, Chun W, editors (2001) Laboratory guide for identification of plant pathogenic bacteria: APS press, Minnesota. 398 p.

[40] De Vos P, Goor M, Gills M, De Ley J (1985) Ribosomal ribonucleic acid Cistron similarities of phytopathogenic Pseudomonas species. Int J Syst Evol Microbiol 35: 169–184.

[41] Stout JD (1980) The role of Protozoa in nutrient cycling and energy flow. Adv Microbial Ecol 4: 1–50.

[42] Jastrow JD, Amonette JE, Bailey VL (2007) Mechanisms controlling soil carbon turnover and their potential application for enhancing carbon sequestration. Clim Change 80: 5–23.

[43] Bailey VL, Smith JL, Bolton Jr H (2002) Fungal∶bacterial ratios in soils investigated for enhanced C sequestration. Soil Biol Biochem 34: 997–1007.

[44] Strickland MS, Rousk J (2010) Considering fungal: bacterial dominance in soils - Methods, controls, and ecosystem implications. Soil Biol and Biochem 42: 1385–1395.

