# Supplementary material for: Soil Microbial Substrate Properties and Microbial Community Responses under Irrigated Organic and Reduced-Tillage Crop and Forage Production Systems
Source: PLoS One. 2014 Aug 4;9(8):e103901. doi: 10.1371/journal.pone.0103901 (PMC4121199; doi:10.1371/journal.pone.0103901)
Supplement: Table S1 — Crop rotations under conventional (CV), organic (OR) and reduced-tillage (RT) management systems in crop and forage production. (DOCX) [file pone.0103901.s003.docx]

Table S1. Crop rotations under conventional (CV), organic (OR) and reduced-tillage (RT) management systems in crop and forage production.

| System | |  | Year |  |  | Winter grazing |
| --- | --- | --- | --- | --- | --- | --- |
|  |  | 2009 | 2010 | 2011 | 2012 |  |
| Crop | CV | Pinto bean | Corn | Sugar beet | Corn | No winter grazing |
|  | OR | Alfalfa | Alfalfa | Corn | Pinto bean |  |
|  | RT | Pinto bean | Corn | Sugar beet | Corn |  |
| Forage | CV | Alfalfa/ grasses | Alfalfa/ grasses | Alfalfa/ grasses | Corn | Cattle grazed during winter 2011/2012 |
|  | OR | Alfalfa/ grasses | Alfalfa/ grasses | Alfalfa/ grasses | Corn |  |
|  | RT | Alfalfa/ grasses | Alfalfa/ grasses | Alfalfa/ grasses | Corn |  |
