# Supplementary material for: Soil Microbial Substrate Properties and Microbial Community Responses under Irrigated Organic and Reduced-Tillage Crop and Forage Production Systems
Source: PLoS One. 2014 Aug 4;9(8):e103901. doi: 10.1371/journal.pone.0103901 (PMC4121199; doi:10.1371/journal.pone.0103901)
Supplement: Table S2 — Timing of management practices by under conventional (CV), organic (OR), and reduce-tillage (RT) management systems on crop and forage production (2009–2012). (DOCX) [file pone.0103901.s004.docx]

Table S2. Timing of management practices by under conventional (CV), organic (OR), and reduce-tillage (RT) management systems on crop and forage production (2009-2012).

| Date | Activity | Management approach | | | | | |
| --- | --- | --- | --- | --- | --- | --- | --- |
|  |  | Forage system | | | Crop system | | |
|  |  | CV | OR | RT | CV | OR | RT |
| Before 2009 | Continuous corn, chemical fertility management | x | x | x | x | x | x |
| **2009** | | | | | | | |
| 04-15 | Tillage with spinner MB plow | x | x |  | x | x |  |
| 04-30 | Tillage with cultivator packer | x | x | x | x | x | x |
|  | Tillage with spinner MB plow | x | x |  | x | x |  |
| 05-05 | Tillage with spinner MB plow | x | x |  | x | x |  |
| 05-07 | Tillage with cultivator packer | x | x | x | x | x | x |
| 05-08 | Fertilizer N: P @ 25: 51 kg ha^-1^ | x |  |  |  |  |  |
|  | Landstar tillage |  |  | x |  |  | x |
| 05-11 | Tillage with cultivator packer |  |  |  |  | x |  |
| 05-12 and 13 | Alfalfa/grass planting | x | x | x |  | x |  |
| 05-14 | Irrigation @ 6.5-mm each day | x | x | x | x | x | x |
| 05-18 | Irrigation @ 6.5-mm each day | x | x | x | x | x | x |
| 05-27 | Fertilizer N: P: S @ 34: 56: 22 kg ha^-1^ |  |  |  | x |  | x |
| 06-01 | Pinto bean planting |  |  |  | x |  | x |
| 06-20 | Irrigation @ 33-mm |  |  |  |  |  |  |
| 06-25 | Pinto bean cultivation |  |  |  | x |  | x |
| 07-03 | Irrigation @ 51-mm | x | x | x | x | x | x |
| 07-08 | Irrigation @ 22-mm | x | x | x | x | x | x |
| 07-25 and 26 | Alfalfa and alfalfa/grass harvest | x | x | x |  | x |  |
| 07-27 | Irrigation @ 13-mm | x | x | x | x | x | x |
| 07-31 | Irrigation @ 15-mm | x | x | x | x | x | x |
| 08-02 | Irrigation @ 18-mm | x | x | x | x | x | x |
| 09-01 and 08 | Bean harvest |  |  |  | x |  | x |
| 09-10 | Irrigation @ 20-mm | x | x | x | x | x | x |
| 09-12 | Irrigation @ 26-mm | x | x | x | x | x | x |
| 09-17 | Alfalfa and alfalfa/grass harvest | x | x | x |  | x |  |
| **2010** | | | | | | | |
| 03-30 | Composted cattle manure @ 12 Mg ha^-1^ |  | x |  |  | x |  |
| 04-12 | Tillage (2 times) with disc plow |  |  |  | x |  |  |
| 04-14 | Fertilizer N: P: S @ 157: 56: 22 kg ha^-1^ | x |  | x | x |  | x |
| 04-21 | Fertilizer N @ 34 kg ha^-1^ |  |  |  | x |  |  |
| 04-28 | Herbicide (Honcho-1 qt/acre; Hell Fire - 2 qts/100 gallons water |  |  |  | x |  |  |
| 04-28 | Planting sugar beet |  |  |  | x |  | x |
| 05-28 | Irrigation @ 18-mm | x | x | x | x | x | x |
| 06-03 | Irrigation @ 18-mm | x | x | x | x | x | x |
| 06-23 | Alfalfa or alfalfa/grass hay harvest |  | x |  | x | x | x |
| 07-02 | Fertilizer N @ 34 kg ha^-1^ in CV; 67 kg ha^-1^ in RT |  |  |  | x |  | x |
|  | Tillage with cultivator |  |  |  | x |  | x |
| 07-16 | Irrigation @ 18-mm | x | x | x | x | x | x |
| 07-26 | Irrigation @ 31-mm | x | x | x | x | x | x |
| 07-30 | Irrigation @ 33-mm | x | x | x | x | x | x |
| 07-03 and 04 | Alfalfa or alfalfa/grass hay harvest | x | x | x |  | x |  |
| 08-15, 18 and 22 | Irrigation @ 13-mm | x | x | x | x | x | x |
| 08-24 | Irrigation @ 13-mm | x | x | x | x | x | x |
|  | Corn harvest |  |  |  | x |  | x |
| 09-08 | Irrigation @ 13-mm | x | x | x | x | x | x |
| 12-03 | Shredding of corn stalks |  |  |  | x |  | x |
| **2011** | | | | | | | |
| 04-25 | Manure @ 67 Mg ha^-1^ |  |  |  |  | x |  |
|  | Fertilizer N: P: S @135: 56: 22 Mg ha^-1^ | x |  | x |  |  |  |
| 04-28 | Fertilizer N: P: S @ 141: 112: 36 in CV; 141: 114: 45 kg ha^-1^ in RT |  |  |  | x |  | x |
| 04-29 | Disc and plow tillage |  |  |  | x | x |  |
| 05-02 | Fertilizer N: P: S @ 134: 67: 22 and pack |  |  |  | x |  |  |
|  | Disc plow tillage and pack |  |  |  |  | x |  |
| 05-03 | Strip tillage |  |  |  |  |  | x |
| 05-04 | Planting sugar beet |  |  |  | x |  | x |
| 05-05 | Compost @ 11 Mg ha^-1^ and corn planting |  | x |  |  |  |  |
| 05-17 | Irrigation @ 13-mm | x | x | x | x | x | x |
| 06-06 | Irrigation @ 13-mm | x | x | x | x | x | x |
| 06-14 | Hay harvest | x | x | x |  |  |  |
| 06-16 | Tillage with cultivator |  |  |  | x |  |  |
| 06-17 | Irrigation @ 13-mm | x | x | x | x | x | x |
| 06-22 | Tillage with cultivator |  |  |  |  | x |  |
| 07-15 | Side dress N @ 134 kg ha^-1^ |  |  |  |  |  | x |
| 08-04 | Hay harvest | x | x | x |  |  |  |
| 09-23 | Hay harvest | x | x | x |  |  |  |
| 09-14 to 18 | Sugar beet harvest |  |  |  | x |  | x |
| 10-20 | Subsoil tillage in CV and Landstar tillage in RT |  |  |  | x |  | x |
| 11-08 and 10 | Corn harvest and shredding of corn stalks |  |  |  |  | x |  |
| 11-11 | Disk tillage |  |  |  |  | x |  |
| **2012** | | | | | | | |
| 03-14 to 16 | Manure @ 56 Mg ha^-1^ |  |  |  |  | x |  |
| 03- 20 | Landstar tillage |  |  |  |  | x |  |
| 04-10 | Compost @ 11 Mg ha^-1^ |  | x |  |  |  |  |
| 04-11 | Irrigation @ 20-mm | x | x | x | x | x | x |
| 04-19 | Disk tillage | x | x |  |  |  |  |
| 04-25 | Subsoil tillage | x | x |  |  |  |  |
| 04-26 | Plow tillage and pack |  | x |  |  |  |  |
| 04-30 | Landstar tillage | x |  |  |  |  |  |
| 05-04 and 06 | Irrigation @ 13-mm | x | x | x | x | x | x |
| 05-11 | Broadcast N: P: S @ 67: 112: 23 kg ha^-1^ | x |  |  | x |  |  |
| 05-14 | Landstar |  |  |  | x | x |  |
|  | Planting |  |  | x |  |  | x |
| 05-15 | Planting | x |  |  | x | x |  |
| 05-16 and 21 | Irrigation @ 13-mm | x | x | x | x | x | x |
| 06-01 | Side dress N: P: S @ 112: 112: 23 kg ha^-1^ |  |  | x |  |  | x |
|  | Irrigation @ 13-mm | x | x | x | x | x | x |
| 06-05 | Land star tillage and planting |  | x |  |  |  |  |
| 06-11 | Irrigation @ 13-mm | x | x | x | x | x | x |
| 06-19 | Herbicide | x |  | x | x |  | x |
| 06-22 and 25 | Irrigation @ 13-mm | x | x | x | x | x | x |
| 06-29 | Cultivate |  |  |  |  | x |  |
|  | Irrigation @ 13-mm | x | x | x | x | x | x |
| 07-01 | Irrigation @ 13-mm | x | x | x | x | x | x |
| 07-03 | Cultivate |  | x |  |  |  |  |
| 07-06, 08, 12, 17, 23, 27, and 29 | Irrigation @ 13-mm | x | x | x | x | x | x |
| 08-02 | Irrigation @ 6-mm | x | x | x | x | x | x |
| 08-04 and 06 | Irrigation @ 13-mm | x | x | x | x | x | x |
| 08-15 | Irrigation @ 6-mm | x | x | x | x | x | x |
| 08-17, 19, 21, and 26 | Irrigation @ 13-mm | x | x | x | x | x | x |
| 08-30 | Irrigation @ 5-mm | x | x | x | x | x | x |
| 08-01, 03, 10, and 17 | Irrigation @ 13-mm | x | x | x | x | x | x |
| 09-26 | Bean plowed down |  |  |  |  | x |  |
| 09-27 | Mulch tillage |  |  |  |  | x |  |
|  | Irrigation @ 13-mm | x | x | x | x | x | x |
| 10-02 | Wheat planting |  |  |  |  | x |  |
| 10-05 to 07 | Corn harvest | x | x | x | x |  | x |
