# Supplementary material for: Soil Microbial Substrate Properties and Microbial Community Responses under Irrigated Organic and Reduced-Tillage Crop and Forage Production Systems
Source: PLoS One. 2014 Aug 4;9(8):e103901. doi: 10.1371/journal.pone.0103901 (PMC4121199; doi:10.1371/journal.pone.0103901)
Supplement: Table S3 — Biomarker phospholipid fatty acids used for identifying taxonomic microbial groups. (DOCX) [file pone.0103901.s005.docx]

Table S3. Biomarker phospholipid fatty acids used for identifying taxonomic microbial groups.

| Taxonomic group | PLFA group | Biomarker fatty acids† | References |
| --- | --- | --- | --- |
| Bacteria (total) | Multiple groups | Sum of 14:0, 15:0, 17:0, i14:0, a15:0, i16:0, i17:0, a17:0, i15:0, 16:1ω9, 16:1 ω7, cy17:0, 18:1ω9 | [41,42] |
| Gram positive | Branched PLFAs | Sum of i14:0, i15:0, a15:0, i16:0, i17:0, a17:0 | [41] |
| Gram negative | Cyclopropyl and mono PLFAs | Sum of 16:1ω9, 16:1ω7, cy17:0, 18:1 ω9 | [41] |
| Fungi (total) | Multiple groups | Sum of 16:1ω5, 18:2ω6 | [41,43] |
| AM fungi | Monounsaturated PLFAs | 16:1 ω5 | [41,43] |
| Fungi/ Bacteria ratio | Multiple groups | Fungi (total)/Bacteria (total) | [38] |
| Protozoa | Polyunsaturated PLFAs | 20:4 ω6 | [41] |

†The fatty acids are named as X: YωZ, where X stands for total number of carbon atoms in the chain, Y stands for number of double bonds, and Z stands for the position of double bonds from the methyl end of the chain. The prefixes ‘i’, and ‘a’ indicate iso, anteiso branching and ‘cy’ indicate a cyclopropyl ring structure.
